# Esophageal emergencies: another important cause of acute chest pain

**DOI:** 10.1186/s13244-020-00915-1

**Published:** 2020-10-09

**Authors:** Venkat Masarapu, Eva Xia, Hongju Son

**Affiliations:** Department of Radiology, Einstein Medical Center Philadelphia, 5501 Old York Road, Philadelphia, PA 19141 USA

**Keywords:** Esophagus, Acute chest pain, Emergency, CT, Fluoroscopy

## Abstract

A variety of esophageal pathologies can present emergently with a chief complaint of acute chest pain. Computed tomography (CT) is often the first line of imaging in esophageal emergencies and provides useful information—even without an initial suspicion—when used in conjunction with other imaging modalities such as esophagography and direct visualization. We review various urgent and emergent esophageal disease entities which may manifest as acute chest pain, with an emphasis on CT and ancillary imaging appearances, while discussing management according to their emergency. Radiologists should be familiar with the imaging findings of these esophageal emergencies in order to provide an accurate diagnosis and recommend timely and appropriate management.

## Key points

- Esophageal pathologies such as obstruction, perforation, inflammation, and infection can be one of many potential etiologies for acute chest pain mimicking acute coronary/aortic syndrome.
- CT is a readily available imaging tool that offers accurate and early diagnosis of acute esophageal conditions in the setting of chest pain even without an initial suspicion.
- Recognizing CT findings of a variety of acute esophageal conditions allows radiologists to recommend timely and appropriate patient management.

## Introduction

Chest pain is the second most common presentation to the emergency department (ED) and accounts for approximately 8–10 million (5–10%) ED visits per year [1]. There are many potential etiologies for acute chest pain ranging from life threatening acute coronary and aortic syndromes to various gastrointestinal pathologies, which account for 7–42% of non-cardiac chest pain ED discharges [2]. Esophageal conditions such as obstruction, perforation, inflammation, and infection are common causes of presentation to the ED, and a presentation of chest pain in the absence of direct trauma should include esophageal disease as a differential diagnosis [3]. The esophagus has traditionally been examined in great detail by contrast fluoroscopy or endoscopy. With the advent of CT as a readily available ancillary tool that offers accurate and early diagnosis of life-threatening conditions (i.e CT triple rule out), it poses an opportunity to evaluate acute esophageal conditions that may be causes of noncardiac chest pain [4]. Therefore, recognition of the appearances of various esophageal pathologies with the potential to lead to emergent presentation is required. This review will describe normal anatomy and radiographic appearance of the esophagus, and classify acute esophageal etiologies into conditions requiring emergent surgical intervention, gastrointestinal intervention, and conditions amenable to medical management (Table 1).

**Table 1:** Management of acute esophageal pathologies

Surgical	• Uncontained esophageal perforation • Fistulas (i.e., aorto-esophageal, pericardioesophageal) • Failed endoscopic foreign body retrievals
Therapeutic esophagogastroduodenoscopy	• Esophageal perforation/lacerations—non surgical candidates • Foreign body ingestion (sharp objects, corrosive battery) and food impaction
Medical management	• Esophageal mucosal lacerations (Mallory-Weiss) • Intramural dissection and hematoma • Infections • Motility disorders

### Anatomy of the esophagus and its relationship to adjacent structures

The esophagus is a muscular tube, 18–26 cm long in adults, with four layers—the mucosa, submucosa, muscularis propria, and adventitia [5, 6]. Throughout the esophagus, there are three natural sites of narrowing: (1) at the level of the cricoid cartilage (at C6, upper esophageal sphincter), (2) at the level of the aortic arch and left mainstem bronchus (at T4/5) from compression by these structures, and (3) at the esophageal hiatus (at T10, lower esophageal sphincter) [7]. In contradistinction to the rest of the gastrointestinal tract, the esophagus lacks a serosa, which more easily allows esophageal pathologies to affect adjacent mediastinal structures including the trachea, pleura, lungs, aorta, and pericardium [8, 9]. The esophagus receives segmental arterial blood supply which divides the organ into cervical, thoracic, and abdominal esophageal regions [5]. The upper third of the esophagus is composed of striated (voluntary) muscle fibers, whereas the lower two thirds are composed mainly of smooth (involuntary) muscle fibers.

#### Cervical esophagus

The cervical esophagus begins at the level of C6, posterior to the larynx, trachea, and cricoid cartilage, and enters the thorax at the level of the sternal notch. Two paired branch vessels of the inferior thyroid artery supply the cervical esophagus, which drains through the inferior thyroid veins to the brachiocephalic veins.

#### Thoracic esophagus

From the thoracic inlet to the aortic arch, the esophagus is slightly left of midline with the trachea anterior and slightly to the right. At the level of the aortic arch, the esophagus remains midline with the trachea anterior and slightly to the right, and the descending aorta on its left. At the carina, the airway is directly anterior to the esophagus and the lung makes intimate contact, especially on the right. The azygos vein lies to the right, posterolateral to the esophagus. The descending aorta lies posteriorly on the left. As the esophagus descends to the mid mediastinum, it lies directly behind the left main stem bronchus and courses down posterior to the left atrium. In the lower mediastinum, the esophagus courses to the left of midline as it enters the diaphragmatic hiatus at the level of T10. Esophageal branches of the thoracic aorta supply the thoracic esophagus, which drains through the azygos vein to the superior vena cava.

#### Abdominal esophagus

From the esophageal hiatus, the esophagus is continuous with the cardia of the stomach at the gastroesophageal junction and lies anterior to the descending aorta left of midline. Esophageal branches of the left gastric artery supply the abdominal esophagus, which drains into the left gastric vein to the portal vein and plays a role as a site of portosystemic collateral pathway.

### Normal radiographic appearance of the esophagus

#### Computed tomography

CT is an excellent first line imaging modality for evaluating the esophagus in the emergent setting. It is fast and readily available, and offers the highest spatial resolution for assessing the extent of esophageal injuries and surrounding mediastinum. The esophagus is partially visualized on neck, abdomen, and thoracic spine CT and the thoracic esophagus is fully identified on chest CT. Administration of oral contrast in the acute setting is not standardized practice, but can potentially elucidate esophageal wall injury with the presence of extraluminal contrast material. If suspicion of esophageal pathology is raised on an initial unenhanced study, a CT esophagography can be performed in patients too sick for fluoroscopic evaluation [10]. In this setting, CT from the thoracic inlet to the diaphragm can be performed after quickly administering an approximately 50 mL solution containing 10% intravenous iodinated contrast material, water, and effervescent granules.

The normal esophageal wall thickness on CT varies depending on anatomical location, degree of distension, and sex, and ranges from 1.9 to 5.68 mm with the thickest area in the abdominal esophagus during contraction [11]. While the esophagus is decompressed in the majority of studies, a normal air column can measure up to 10 mm in transverse dimension, and up to 15 mm in the segment of esophagus between the cardiac ventricles and gastroesophageal junction [12]. Although the degree of tissue delineation is better appreciated with increased mediastinal fat, the margins of the outer esophageal wall, thoracic aorta, and azygos veins should nevertheless be clearly delineated, with any indistinctness in these margins suggesting underlying mediastinal pathology [13].

#### Contrast esophagography

While CT is the most readily available modality for esophageal emergencies, contrast esophagography has utility as a highly sensitive imaging test for many esophageal pathologies [14]. In the emergent setting with suspected esophageal laceration or perforation, a single contrast study with water-soluble contrast can be performed. Spot radiographs should be obtained in multiple planes, and evaluation of all regions of the esophagus should be performed with the smallest field of view in order to obtain the best spatial resolution. The normal esophagus is smooth and featureless without intraluminal filling defects or extraluminal contrast extravasation.

#### Magnetic resonance imaging

Magnetic resonance imaging (MR) has its role in the evaluation of the esophagus, particularly in tumor staging, surgical planning, or nonionizing dynamic functional studies [15, 16]. However, MR is not routinely utilized in the acute setting, and description of the MR appearance of esophageal pathologies lies outside of the scope of this article.

## Surgical emergency

### Esophageal perforation

**Fig. 1:**
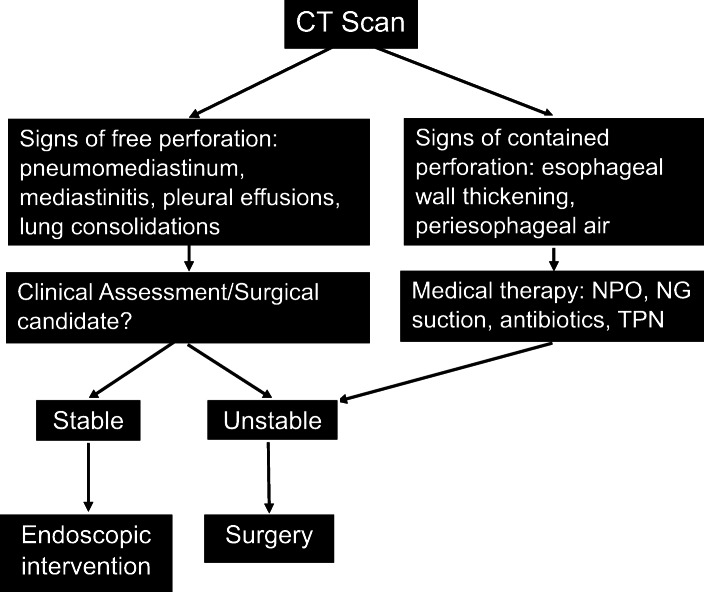
Clinical consideration for esophageal perforation. Note. NPO = nothing by mouth, NG = nasogastric, TPN = total parenteral nutrition

Esophageal perforation (EP) is a rare but morbid entity associated with a mortality rate of 20–40%, with higher mortality seen with delayed presentation [17, 18]. Symptoms are nonspecific depending on the location of the perforation and patients may present with dysphagia, neck pain or crepitus, chest pain, or epigastric pain. The most common cause of EP is iatrogenic from endoscopic instrumentation or thoracic surgery, and accounts for 59% of cases [19, 20]. Other etiologies include spontaneous perforation, corrosive or sharp foreign body ingestion, trauma, and malignancy (Table 2). Management is variable and takes into account the severity and location of the perforation as well as sepsis status and damage to surrounding structures. Surgical consultation is always merited and the preferred approach is surgical primary repair (Fig 1) [21, 22]. However, distinction of a contained perforation such as a thoracic EP contained without drainage into the pleural space is important as the treatment for small contained perforations can be non-surgical and patients may benefit from stent placement or medical management, avoiding invasive surgery [23].

**Table 2:** Causes of esophageal perforation

Iatrogenic	• Fundoplication and esophageal myotomy • Cardiac ablation, transesophageal echocardiography • Thoracic surgery, anterior cervical discectomy
Spontaneous	• Increased intraluminal pressure from retching or forceful vomiting (i.e. Boerhaave syndrome)
Foreign Body	• Sharp or caustic materials • Impaction causing wall ischemia
Trauma	• Penetrating or blunt trauma
Malignancy	• Primary or metastasis

#### Iatrogenic and idiopathic esophageal perforation

**Fig. 2:**
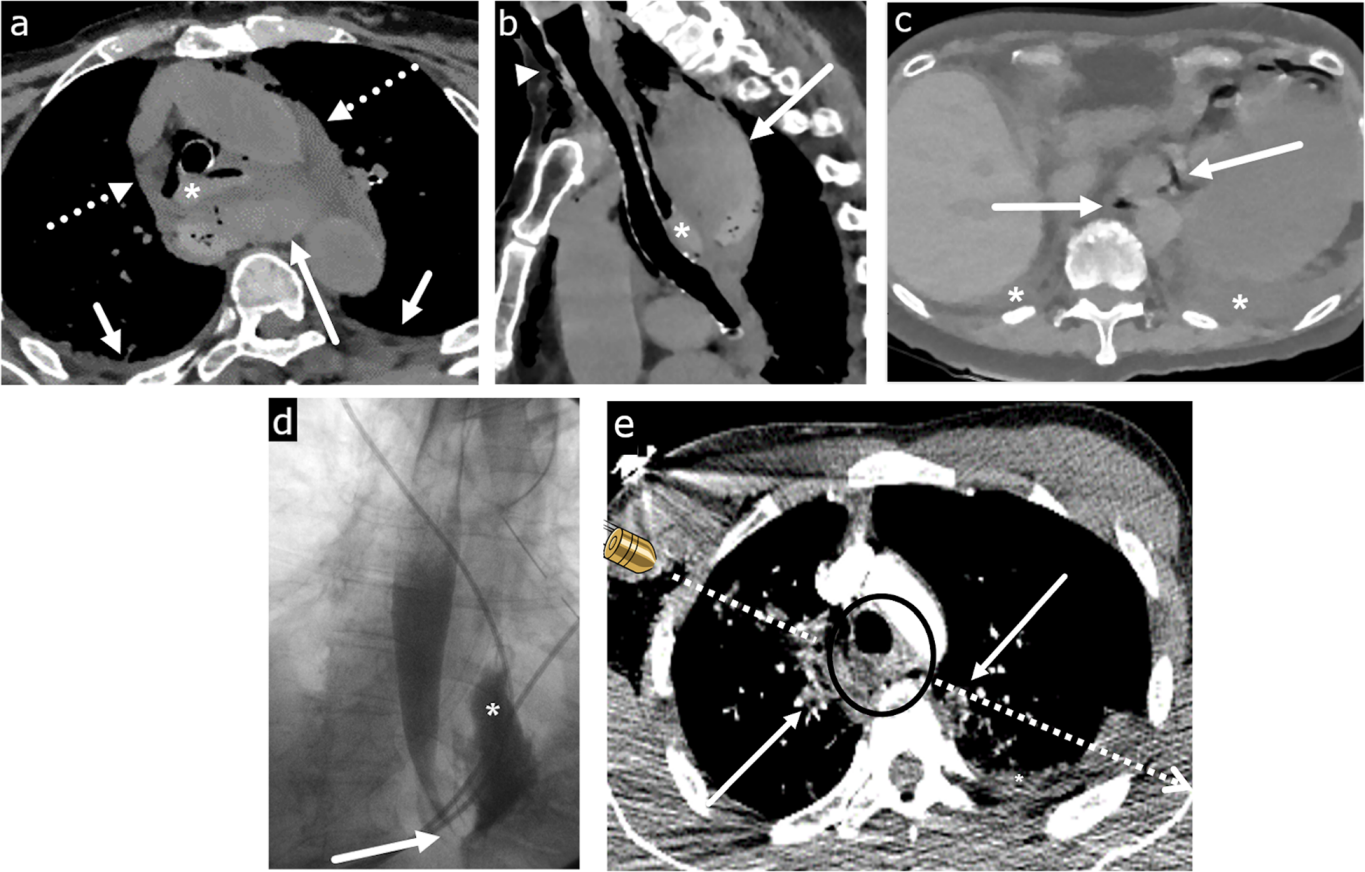
Esophageal perforation. **a**, **b** Iatrogenic esophageal perforation. Eighty-year-old woman with chest pain after transesophageal echocardiography. Axial (**a**) and sagittal (**b**) CT images show a dilated and blood-filled upper esophagus (asterisk) contiguous with a large posterior hematoma containing multiple locules of gas (long arrow). Extensive hemo-pneumomediastinum (dashed arrow), small bilateral pleural effusions (short arrow), and subcutaneous emphysema in the lower neck (arrowhead) are seen. **c, d** Boerhaave syndrome. Eighty-seven-year-old woman with epigastric and back pain after forceful vomiting. **c** Axial CT image at the gastroesophageal junction shows extraluminal gas surrounding the lower esophagus (arrow). Bilateral pleural effusions (asterisk). **d** Single contrast esophagography demonstrates luminal narrowing and irregularity of lower esophagus (arrow) with large contrast extravasation into the left pleural space (asterisk). **e** Traumatic esophageal perforation. Twenty-five-year-old male with gunshot wound to the chest. Axial CT image shows a bullet tract from right axilla to the left upper hemithorax (dashed arrow) resulting in bilateral pneumothoraces (not shown), left hemothorax (asterisk), and pulmonary contusion and laceration in the bilateral upper lobes (arrow). There is esophageal thickening and indistinctiveness with a small hematoma (circle) representing esophageal injury/perforation

In patients presenting with acute chest pain after a recent history of instrumentation such as simple endoscopy, esophageal stricture dilation, anterior cervical discectomy, or various cardiac procedures such as transesophageal echocardiography or radiofrequency catheter ablation, careful evaluation should be made for the diagnosis of EP [24, 25]. CT findings may be subtle and can include small locules of periesophageal and mediastinal air or fluid, left pleural effusion, or focal wall thickening (Fig. 2a, b). Similar findings can also be seen in spontaneous esophageal perforation such as Boerhaave syndrome, which is induced from a sudden increase in intraluminal pressure from acts such as forceful vomiting, cough, or Valsalva maneuver (Fig. 2c, d). Spontaneous perforation classically results in a longitudinal tear along the posterior wall of the abdominal esophagus, whereas the hypopharynx is the most commonly affected site in iatrogenic perforation [26, 27]. In a patient with suspected EP who is otherwise clinically stable, contrast esophagography with water-soluble contrast can be performed first to evaluate for extraluminal contrast material, but if negative, should be followed by barium esophagography due to the higher sensitivity of barium in the detection of EP [28]. In the authors’ experience, an EP is best visualized with the patient in the left posterior oblique position.

#### Traumatic esophageal perforation

**Fig. 3:**
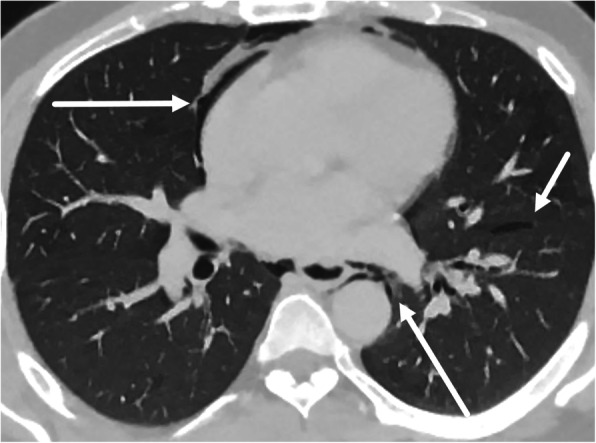
Pneumomediastinum. Thirty-four-year-old male with chest pain after vigorous cough. Axial CT image shows moderate pneumomediastinum (long arrow) caused by Macklin effect. Subpleural gas (short arrow) caused by alveolar rupture in the setting of increased intrathoracic pressure with air dissecting along the subpleural interstitium, interlobular septa, peribronchovascular interstitial sheaths, and eventually into the mediastinum (Macklin effect). While this mimics EP, there is a lack of inflammatory changes and hydropneumothorax commonly associated with esophageal perforation

Due to its posterior and relatively protected location, direct injury to the esophagus is rare but results in great morbidity from associated synchronous airway, aortic, or spinal injuries [29, 30]. In the neck, however, the cervical esophagus and upper thoracic esophagus can be particularly susceptible to penetrating trauma [31]. Although fluoroscopy is the most sensitive study for EP, CT is often the first line of imaging in the emergent setting. Since the esophagus is usually collapsed, the role of CT is to assess for the secondary findings of EP including pneumomediastinum, posterior mediastinal hematoma, and pleural effusion (Fig. 2e). The presence of multiple of these supporting findings in conjunction with esophageal wall thickening, extraluminal contrast material, or focal wall defect should lead to a high index of suspicion for traumatic EP. A potential pitfall for misdiagnosis of EP in the setting of trauma is the Macklin effect, which also demonstrates pneumomediastinum, but develops secondary to increased intrathoracic pressure from alveolar rupture [32]. Air subsequently dissects along the peribronchovascular interstitial sheaths, interlobular septa, and into the mediastinum (Fig. 3). However, the Macklin effect is not associated with pleural effusion or the reactive changes associated with esophageal wall perforation.

#### Malignancy induced esophageal perforation

**Fig. 4:**
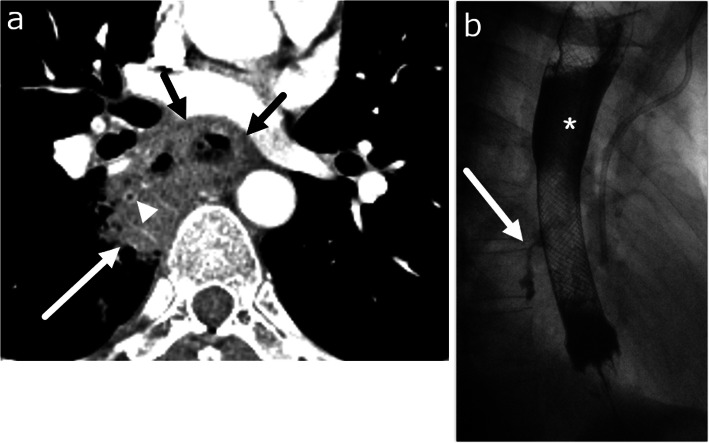
Malignancy-induced esophageal perforation and esophago-bronchial fistula. Forty-nine-year-old male with recently diagnosed esophageal cancer presenting with chest pain. **a** Axial CT image shows circumscribed mass of the lower esophagus consistent with esophageal cancer (short black arrow). The esophagus is perforated into the right lower lobe containing debris and gas (white long arrow). The medial basal segment bronchus is encased by the debris (arrowhead). An esophageal stent was urgently placed. **b** Esophagography obtained 2 weeks after placing the esophageal stent shows an esophago-bronchial fistula (arrow). Appropriate position of esophageal stent (asterisk) is seen

Malignancy-related esophageal perforations can result from primary late-stage esophageal cancer, iatrogenic perforation from endoscopic treatment of esophageal cancer, or radiation induced esophageal injury leading to perforation [33]. Once EP occurs, morbidity and mortality significantly increase due to risk of infection such as mediastinal or lung abscess, or development of esophageal-tracheal fistula (Fig. 4) [34].

#### Esophageal fistulas

**Fig. 5:**
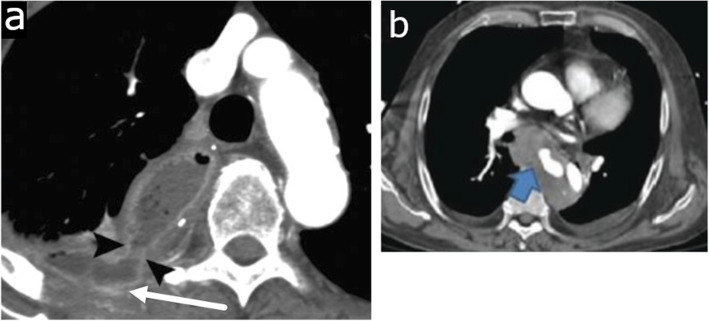
Esophageal fistulas. **a** Esophago-pleural fistula in a 61-year-old male with squamous cell carcinoma of mid esophagus, status post esophagectomy and gastric pull through presenting with fever. Axial CT image obtained 12 days after surgery shows a fistula (arrowheads) between the gastric tube and the right pleural space. Fluid collection with pleural enhancement and thickening (arrow) is suspicious for empyema. Reprinted with permission from Kim et al. Radiographics 2007; 27(2):409-429. **b** Aorto-esophageal fistula in a 70-year-old male patient with hypertension and regular alcohol use presenting with hematemesis and melena. Axial image of CT chest angiography shows type B aortic dissection with rupture, communication, and extravasation between aorta and esophagus (blue arrow) in the arterial phase. Reprinted from Kokatnur. Indian J Crit Care Med. 2015;19(2):119–121

**Fig. 6:**
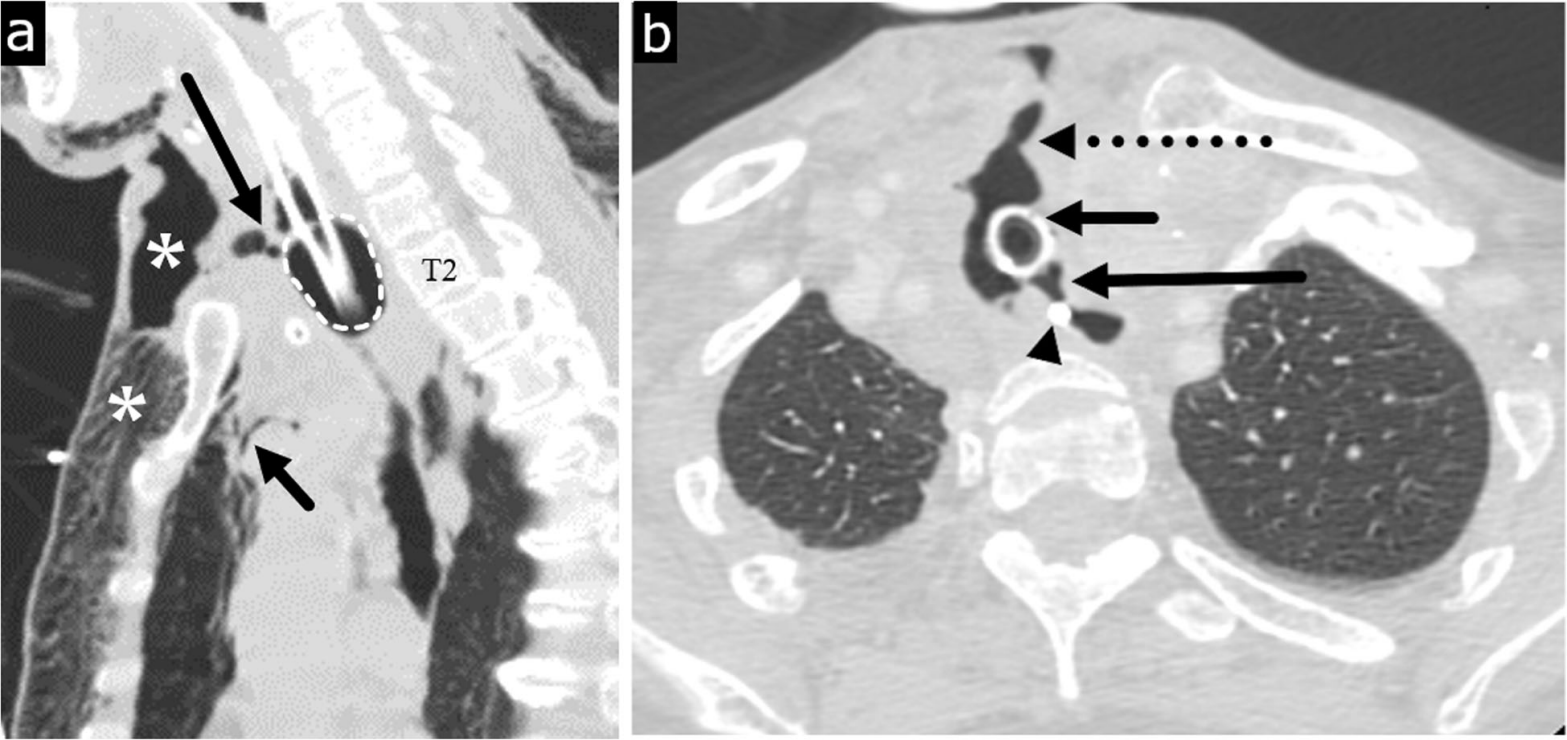
Tracheoesophageal fistula. Ninety-one-year-old female with long term intubation with subcutaneous crepitus. **a** Sagittal CT image shows a traumatic anterior tracheal wall defect (long black arrow) caused by an overinflated endotracheal tube balloon (dotted circle) resulting in a tracheocutaneous fistula and severe anterior chest wall subcutaneous emphysema (asterisks). Small pneumomediastinum (short black arrow) is seen. **b** Axial CT image obtained 3 weeks later shows a new tracheoesophageal fistula in the region of the previous overinflated endotracheal tube balloon (long black arrow), and a persistent tracheocutaneous fistula (dashed black arrow). Tracheostomy tube (short black arrow). Enteric tube (arrowhead)

Transmural injury to the esophagus or prolonged inflammation of the mediastinum can have lasting effects, and due to the lack of serosa, the esophagus can be vulnerable to forming various fistulas with surrounding structures including the trachea or bronchus, pleura, pericardium, and rarely the aorta (Figs. 4 and 5) [35, 36]. The most common of these is development of a tracheoesophageal fistula (TEF), an abnormal connection typically between the anterior wall of the esophagus and posterior wall of the airway. Formation of a TEF can occur during the healing process after various insults such as mucosal injury of the esophageal and tracheal walls in blunt trauma, necrosis from tumor erosion, or focal ischemia as a result of an overinflated endotracheal cuff (Fig. 6). The classic clinical sign of TEF is paroxysmal cough and choking after swallowing liquids [37, 38]. When clinically suspected, esophagography is the study of choice with its ability to demonstrate tracheoesophageal communication of contrast. CT findings can include the presence of a fistulous tract extending from the esophagus to the bronchial tree, or extraluminal contrast. On CT, while water soluble oral contrast can more easily define smaller fistulous tracts, it is not always required. Associated findings such as inflammation and air tracking abnormally in a non-anatomic orientation can serve as diagnostic guides.

Fistulous tracts from the esophagus to additional adjacent structures are typically a result of prior surgery or endoscopic procedure, invasion of esophageal carcinoma, or sequala of prior radiation therapy. In the setting of esophagopleural fistulas, radiography can demonstrate a pleural effusion or hydropneumothorax. On CT, delineation of the fistula tract itself, or the presence of pleural air and fluid or oral contrast agent if administered are contributory findings to accurate diagnosis. In esophagopericardial fistulas, CT can identify pericardial air, indicating the anomalous tract. Aorto-esophageal fistulas are especially rare and typically form in the setting of prior thoracic aorta repair or eroding or ruptured thoracic aortic aneurysm [39, 40]. In stable patients in whom esophageal pathology is suspected, esophagography will demonstrate indentation or deviation of the esophagus secondary to the aneurysm and possible mucosal ulceration or spill of contrast posteriorly into the aorta. CT findings of oral contrast extravasation into the aorta, periaortic or intraluminal gas, and focal esophageal wall thickening are highly suggestive of the fistula. Spontaneous closure of esophageal fistulas is rare and management depends on etiology, size, anatomy, and additional patient comorbidities [41]. Esophageal stenting can serve as a palliating treatment in patients too ill for surgical repair, which is the preferred treatment option [42, 43].

## Gastrointestinal emergency

### Foreign body (FB) ingestion and impaction

**Fig. 7:**
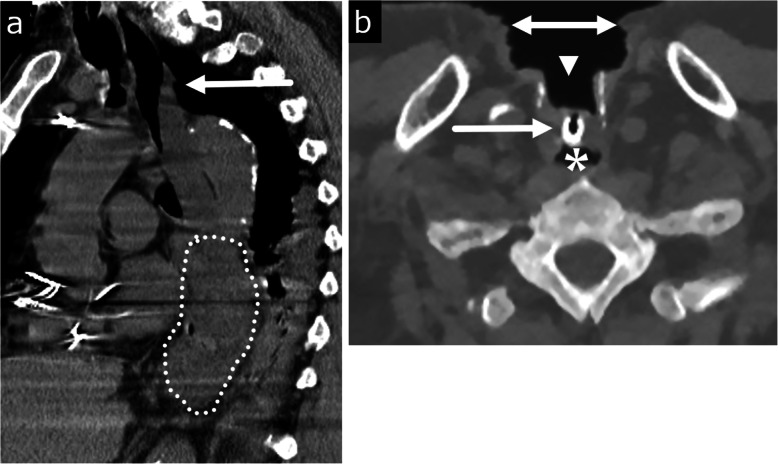
Esophageal obstruction with food bolus impaction (**a**) and therapeutic foreign body/prosthesis (**b**). **a** An 83-year-old demented male with chest pain during meal. Sagittal CT image shows a large chunk of food impaction in the mid to lower esophagus (dotted area). The upper esophagus is mildly dilated with an air-fluid level. **b** A 71-year-old male with history of laryngectomy for laryngeal cancer with a tracheoesophageal voice prosthesis which may mimic a foreign body. Axial CT image shows a small tubular shaped radiopaque device (arrow) between the tracheostomy (arrowhead) and the upper esophagus (asterisk). There is a tracheostomy defect and associated large skin defect anteriorly (bidirectional arrow)

In the adult population, food bolus is the most common esophageal FB and most commonly occurs in elderly or mentally impaired adults (Fig. 7a) [44]. Non-food bolus FB ingestions such as bones, toothbrushes, and razor blades can occur accidentally among denture users, or intentionally among children, incarcerated adults, and patients with psychiatric disorders [45]. Foreign body obstruction and food impaction in adults usually occurs in the esophagus at the physiologic sites of narrowing—the cricopharyngeal cartilage, in the midthorax posterior to the aortic arch and left mainstem bronchus, and at the diaphragmatic hiatus [46].

Patients with FB ingestions and food bolus impactions can present with retrosternal pain, dysphagia, and odynophagia. The CT appearance of various foreign bodies depends on the composition of the ingested product. Large radiopaque materials such as batteries and metal can be easily identified on radiograph and CT, but an awareness of medical devices such as transesophageal voice prostheses is required so as not to be mistaken for an abnormal FB (Fig. 7b). Although 80–90% of ingested FBs are able to pass spontaneously, endoscopic intervention is needed in 10–20% of patients [47, 48]. According to clinical guidelines from the American Society for Gastrointestinal Endoscopy and European Society of Gastrointestinal Endoscopy, emergent therapeutic endoscopic intervention (within 2–6 h) is indicated for (1) FBs causing complete esophageal obstruction resulting in an inability to tolerate secretions and (2) sharp-pointed objects and disk batteries, due to the increased risk of perforation, mucosal damage, and bleeding [49–52]. Retrospective studies have shown that delayed therapeutic endoscopy, greater than 1 day, in patients with sharp FB impactions results in increased complications, especially among the elderly [46]. Urgent endoscopic intervention (within 24 h) is indicated for partial esophageal obstruction, and emergent surgical intervention is required in patients with esophageal perforation or failed endoscopic retrieval.

## Medical management

### Intramural dissection and hematoma

In the spectrum of esophageal mucosal injuries ranging from mucosal laceration (Mallory-Weiss tear) to full thickness tear (esophageal perforation), intramural esophageal dissection (IED), and intramural hematoma of the esophagus (IHE) are in the intermediate range and refer to pathologies limited to the esophageal wall, akin to aortic dissection and aortic intramural hematoma. The symptoms of IED and IHE also similarly often mimic acute aortic syndromes with the classic triad of chest pain, dysphagia, and hematemesis [53–55]. Common risk factors for the development of these conditions include recent instrumentation, coagulopathy, foreign body ingestion, and vomiting.

**Fig. 8:**
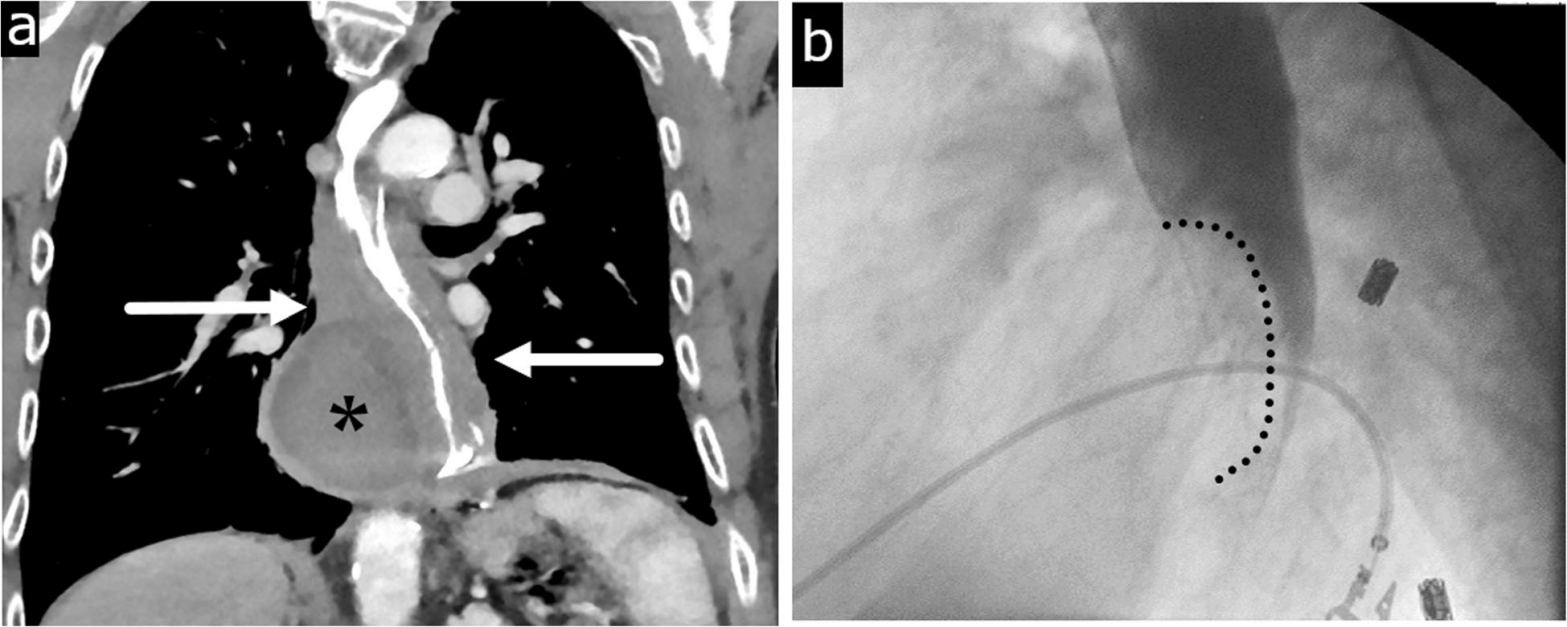
Intramural hematoma. Sixty-one-year-old male with severe chest pain and dysphagia status post recent radiofrequency ablation for Barrett’s esophagus. **a** Coronal CT image shows a smoothly demarcated eccentric homogeneous intramural hematoma (asterisk) in the right wall of diffusely thick-walled esophagus (arrows). **b** Contrast esophagography demonstrates smoothly marginated mass effect (dotted line) on the lower esophagus by the intramural hematoma

IHE is a rare condition characterized by collection of blood within the esophageal wall and can be divided into five subtypes depending on the etiology: traumatic, emetogenic, coagulopathic, aorta-related, and spontaneous [54]. The pathophysiology starts with focal submucosal hemorrhage which dissects along the submucosa and forms a hematoma which may be concentric or eccentric [54]. The most common location is in the lower esophagus due to a lack of adjacent supporting structures. CT is the modality of choice for evaluation of IHE both in its ability to differentiate from an aortic process and to identify the characteristic findings of concentric or nonconcentric esophageal wall thickening and presence of a high attenuation mass with varying degrees of luminal narrowing (Fig. 8a). Fluoroscopic contrast esophagography demonstrates a well circumscribed filling defect corresponding to the hematoma (Fig. 8b).

**Fig. 9:**
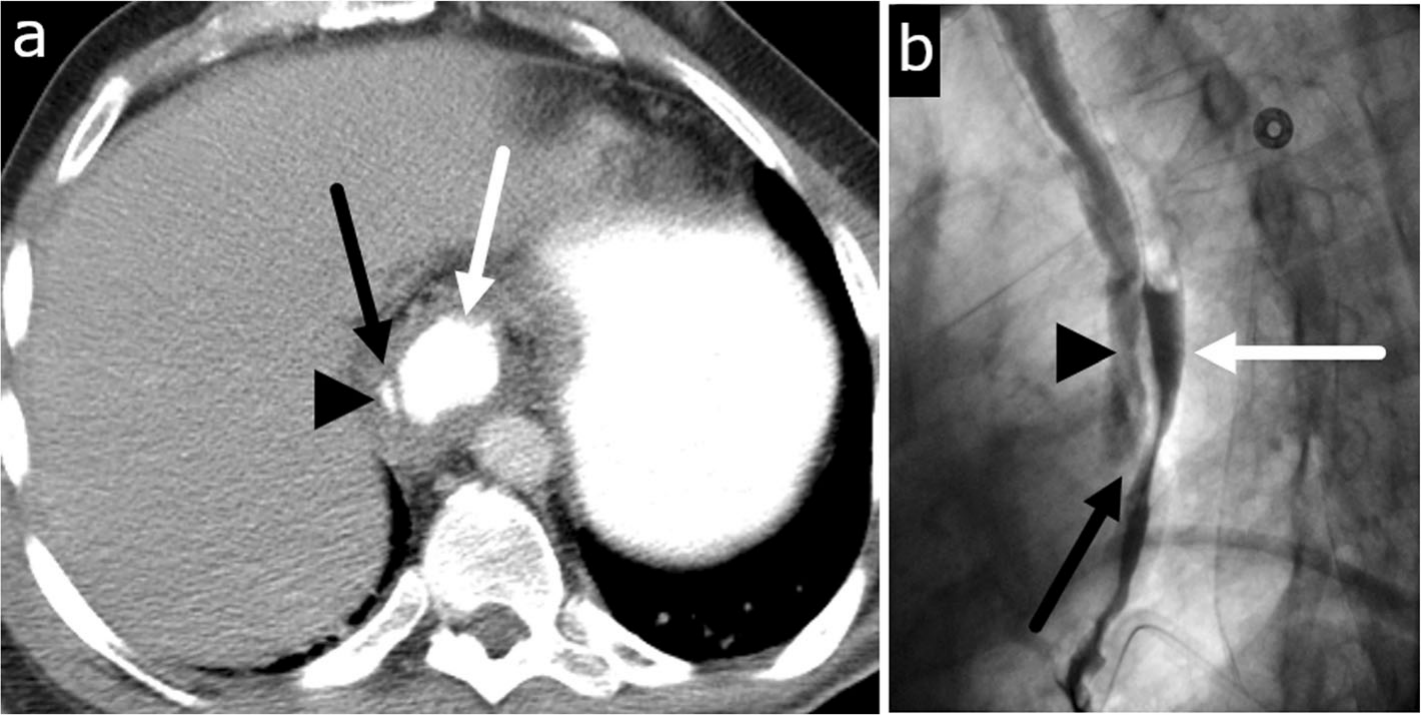
Intramural dissection. Fifty-one-year-old male with chest pain radiating to the back after forceful vomiting. **a** Axial CT image shows an intramural dissection flap (arrow) between the true (arrowhead) and false lumen (white arrow) in the lower esophagus. **b** Barium esophagography shows two parallel lumens and an intervening dissecting flap filling defect (black arrow) giving the esophagus the double barrel appearance. True lumen (arrowhead). False lumen (white arrow)

IED is another entity on the spectrum of mucosal injury in which there is dissection between the mucosa and submucosa without perforation. There are two postulated pathogeneses: (1) a submucosal bleed which tears through the mucosa and decompresses the hematoma and (2) mucosal injury which dissects through the submucosa [53]. Classic CT and fluoroscopic findings include a thin dissection flap between the true and false lumen giving the esophagus a double-barrel appearance (Fig. 9). Prognosis of both IHE and IED is excellent with conservative medical management, and resolution of symptoms usually occurs within 1–3 weeks [56, 57].

### Acute esophagitis

**Fig. 10:**
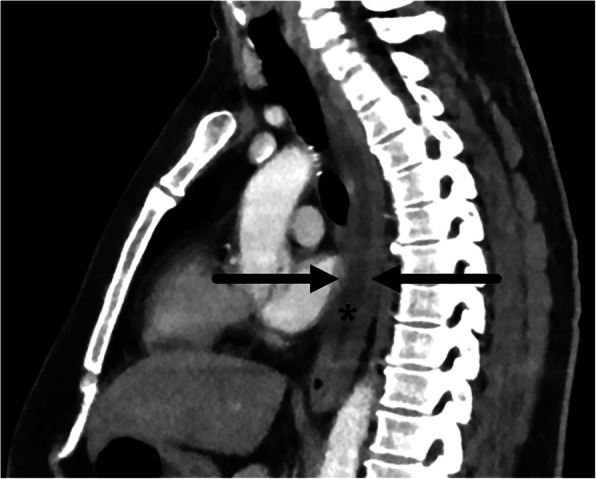
Acute esophagitis. Fifty-year-old male with diffuse chest pain and mild fever. Sagittal CT image show diffuse circumferential wall thickening with mild enhancement involving almost entire esophagus (arrows). Intraluminal fluid (asterisk) is noted

Inflammation of the esophagus can arise from multiple etiologies including gastroesophageal reflux, medication, radiation, or infection [58–61]. Although barium esophagography and endoscopy are more sensitive modalities in the evaluation of esophagitis, patients may present with chest pain and dysphagia in the emergent setting where CT thorax is obtained [62, 63]. CT findings include diffuse circumferential wall thickening (> 5 mm)—which is often nonspecific and may be seen in benign or malignant etiologies—and hyperenhancement of the mucosa relative to hypoenhancement of the submucosa from edema, producing a target sign (Fig. 10) [64]. In the correct clinical setting, these nonspecific CT findings may lead to the diagnosis of esophagitis for the patient’s symptoms. Treatment of esophagitis varies based on the cause, but would include removal of the inciting source, if any, and alleviation of symptoms with conservative management.

### Achalasia

**Fig. 11:**
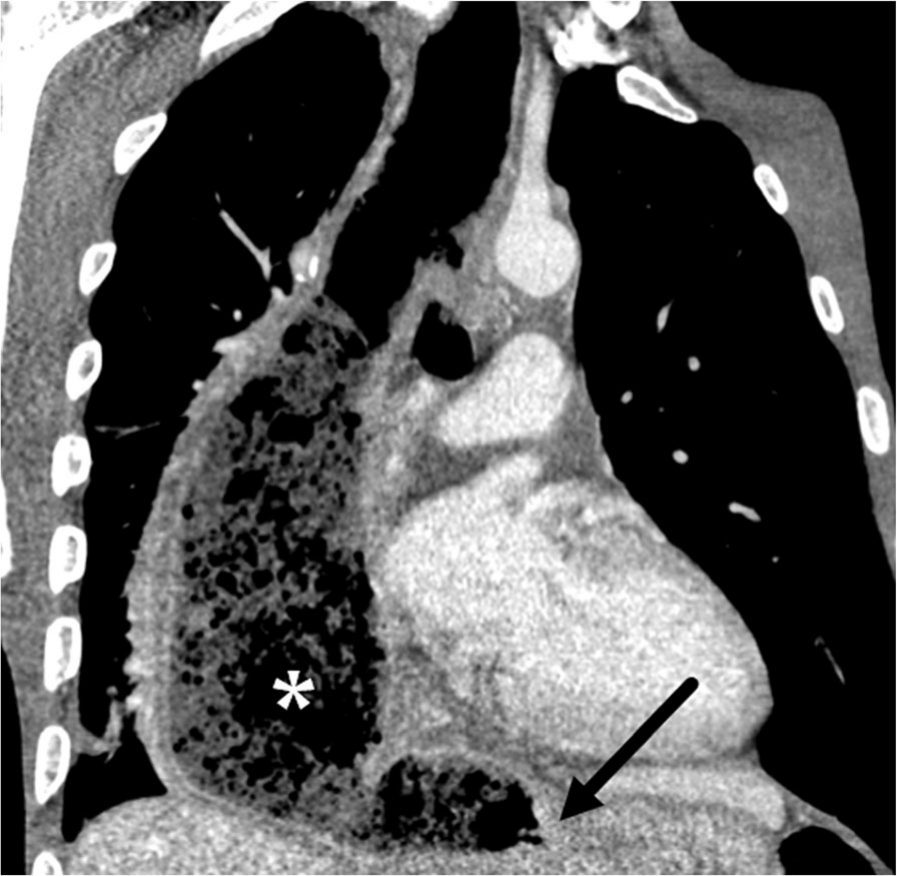
Primary achalasia. Forty-eight-year-old male with dysphagia and weight loss. Coronal CT image shows markedly dilated esophagus with an air-debris level (asterisk). An abrupt stricture (arrow) is seen in the lower esophagus (bird beak sign)

Primary achalasia is an esophageal motility disorder manifested by failure of relaxation of the lower esophageal sphincter and loss of peristalsis of the lower esophagus, resulting in dilation of the esophagus [65]. Men and women are equally affected, with diagnosis typically between the ages of 25 and 60 [66]. The etiology of primary achalasia is unknown, but secondary achalasia can occur in disease such as Chagas disease, amyloidosis, sarcoidosis, neurofibromatosis, MEN type 2B, Sjogren syndrome, and malignancy [67–73]. The most common symptoms are dysphagia and regurgitation of undigested food. Chest pain is usually the presenting symptom in younger patients, but is seen in 40–60% of patients overall. On chest radiography, findings include widened mediastinum with or without a fluid level, and lateral displacement of the trachea. CT findings include a dilated esophagus with fluid/debris level, and in primary achalasia, there is usually no esophageal wall thickening or mass at the cardia (Fig. 11). The presence of focal wall thickening at the site of narrowing may indicate malignancy induced secondary achalasia. Barium swallow esophagography is the imaging study of choice and demonstrates the characteristic bird beak sign and tertiary contractions. Treatment depends on disease severity, and ranges from lifestyle changes to endoscopic interventions such as pneumatic dilatation to surgical myotomy.

## Conclusion

A variety of esophageal pathologies can present emergently with a chief complaint of acute chest pain. CT is often the first line of imaging in esophageal emergencies and provides useful information, even without an initial suspicion, when used in conjunction with other imaging modalities such as esophagography and direct visualization. Radiologists should be familiar with the imaging findings of these esophageal emergencies to provide accurate diagnosis as well as to recommend timely and appropriate management.

## Data Availability

Not applicable.

## Abbreviations

CT: Computed tomography
ED: Emergency department
FB: Foreign body
IED: Intramural esophageal dissection
IHE: Intramural hematoma of the esophagus
MR: Magnetic resonance imaging EP Esophageal perforation
TEF: Tracheoesophageal fistula
